# Rumen microbes affect the somatic cell counts of dairy cows by modulating glutathione metabolism

**DOI:** 10.1128/msystems.01093-24

**Published:** 2025-03-19

**Authors:** Hua Zhang, Tianhang Lu, Shijiao Guo, Tianying He, Min-Kyoung Shin, Chaochao Luo, Jinjin Tong, Yinhua Zhang

**Affiliations:** 1 College of Veterinary Medicine, Beijing University of Agriculture, Beijing, China; 2 Animal Science and Technology College, Beijing University of Agriculture, Beijing, China; 3 Department of Microbiology, College of Medicine, Gyeongsang National University, Jinju, South Korea; 4 College of Life Sciences, Shihezi University 70586 , Shihezi, Xinjiang, China; University of Wisconsin-Milwaukee, Milwaukee, Wisconsin, USA

**Keywords:** Bacteroidetes, glutamate metabolism, somatic cell count, mammary gland, dairy cows

## Abstract

Healthy mammary glands are essential for high-quality milk production in the dairy industry. The relationship between somatic cell counts (SCCs), rumen fermentation, and microbiota interactions remains unclear. This study integrated physiological indicators, high-throughput 16S rRNA gene sequencing, and metagenomics data analysis to investigate the mechanisms linking rumen microbes and mastitis and to evaluate the changes in milk production and serum cytokine levels in cows with low (L-SCC) and high (H-SCC) somatic cell counts. Compared with the L-SCC group, the H-SCC group exhibited significantly lower lactose and fat contents in milk, reduced rumen fermentation product levels, and increased abundances of Bacteroidetes, Firmicutes, *Lachnospiraceae*, *Prevotella*, and Rumiclostridium. Elevated serum levels of IgG2, IgM, IL-1β, IL-6, and TNF-ɑ in the H-SCC group indicated inflammation and rumen microbiota dysbiosis. Functional analysis of microbial communities revealed significant enrichment in pathways related to glutathione metabolism, thyroid hormone synthesis, hypertrophic cardiomyopathy (HCM), the phosphotransferase system (PTS), the P53 signaling pathway, and the Jak-STAT signaling pathway. Correlation network analysis showed that changes in bacterial families, such as *Rikenellaceae, Muribaculaceae,* and *Prevotellaceae*, were associated with cytokines, rumen fermentation, and milk quality. The study highlights the interaction between rumen microbiota homeostasis and mammary gland health, indicating that rumen fermentation status influences serum inflammation and milk quality. Modulating rumen fermentation to enhance mammary gland immune function presents a viable strategy for sustainable dairy industry development with long-lived, highly productive cows.

**IMPORTANCE:**

High somatic cell counts (SCCs) are a key biomarker of mastitis and are associated with decreased milk production and significant economic losses in dairy farming. This study systematically examines the relationship between elevated SCCs, rumen microbial dysbiosis, and host inflammatory responses, shedding light on the intricate interplay between microbial ecosystems and host physiology. The findings highlight the potential for microbiota-targeted interventions to reduce inflammation, improve milk composition, and enhance dairy cow productivity. Rather than presuming a direct causative link between SCC-associated dysbiosis and inflammation, this research focuses on their interdependent dynamics, offering a nuanced understanding of the complex biological mechanisms involved. This work advances knowledge of host-microbiota interactions in livestock, providing practical insights for the development of innovative strategies to manage mastitis and improve overall herd health. By adhering to One Health principles, this study underscores the significance of sustainable agricultural practices that prioritize animal welfare, environmental stewardship, and food security. These findings establish a robust foundation for future research into microbiota-driven solutions aimed at enhancing the health and productivity of dairy cattle.

## INTRODUCTION

Milk with its excellent balance of nutrients is a beneficial dietary addition for human health (1). To satisfy the demand for high-quality dairy products, the development of higher-yielding dairy cows has become increasingly profitable, but some of the regimens, such as starch-rich diets and the overuse of antibiotics, put cows under considerable stress, including increased oxidant levels (2). Thus, improving host immune function and mammary gland health is critical to the milk industry (3). Gazzola et al. (4) found that treatment conditions can predispose animals to pathogen invasion, causing metabolic disruption, inflammatory cytokine increase, and cellular (e.g., neutrophils) damage to the mammary gland. This is also the main reason for the increased SCCs in milk.

However, the health of the udder can be compromised not only by pathogens, oxidant accumulation, and mammary turnover regulation but also by metabolic diseases such as subacute ruminal acidosis and ketosis. Previous studies have shown that gut dysbiosis accelerates the progression of *Staphylococcus aureus-*induced mastitis in mice (5). Moreover, it has been reported that the rumen microbial taxa affect dairy cow milk performance and that rumen microbes and their metabolites can migrate to distal extraintestinal organs, potentially contributing to mastitis (6). However, how bacteria from the rumen are involved in the pathogenesis of mastitis remains unknown.

The rumen is the largest digestive organ, and its microbial community structure and digestive efficiency are the most significant guarantees of the quality and yield of milk from a dairy cow herd. The complex ruminal ecosystem influences the composition and function of the microbiota and plays a central role in the regulation of metabolic functions such as digestion and absorption, and the maintenance of immune homeostasis. Gastrointestinal diseases, such as subacute ruminal acidosis, commonly occur with mastitis in dairy cows (7). An increasing number of studies have revealed that disturbances in the rumen microbiota are accompanied by higher levels of proinflammatory cytokines and acute-phase proteins in the serum and an increased risk of laminitis (8, 9). Our previous study revealed that the microbiomes of rumen and milk samples from dairy cows with subclinical *Streptococcus agalactiae* mastitis contained similar microbial compositions (10), including *Firmicutes* and *Proteobacteria*, suggesting a transfer between the rumen and the mammary gland, similar to that reported for the gut-breast axis in humans from mothers to neonates (11). Zhao et al. (12) demonstrated that sialic acid from the microbiota-gut-mammary axis aggravated mastitis caused by disturbances in the commensal bacteria of the intestine. The transfer of fecal material from cows with mastitis to germ-free mice resulted in a mammary inflammatory response, indicating a close connection between the rumen microbiota and the occurrence and development of mastitis in dairy cows (13). In addition, the relative abundances of *Lachnospiraceae*, *Neisseriaceae*, and *Moraxella* were significantly increased in the rumens of dairy cows with clinical mastitis, which is consistent with the increasing trend of the proinflammatory metabolite leukotriene B4 (14). However, whether the rumen microbes are the drivers or the passengers in the development of mastitis is unknown. The underlying mechanisms and dynamic relationships need further elucidation.

Although exactly how the microbiota mediates host health remains elusive, the functional potential of the rumen microbiome and its relationship with animal performance has been extensively studied through metagenomics and meta-transcriptomics approaches (15, 16). Recently, a fast-emerging sequencing technique has been reported to significantly improve microbial reference genomes and read classification by 50%–70% (17, 18). By assembling complete microbial genomes directly from metagenomic binning, this method provides a deeper understanding of the mechanisms through which the rumen microbiota act. For example, a previous study demonstrated the critical microbial genomes involved in digestion in the rumen by integrating data from metagenomic binning and single-cell RNA sequencing to link microbial genomes and single epithelial cells to nutritional systems (19). Furthermore, using a method combining metagenomic binning and metatranscriptome analysis, Xue et al. found that *Selenomonas* bacteria in the rumen actively interacted with members of the family *Succinivibrionaceae* to significantly influence carbohydrate metabolism (20). Considering all the possible interactions between rumen microbes and host metabolic pathways, we hypothesize that ruminal dysbiosis could facilitate a host inflammatory reaction. In turn, inflammation can cause a high SCC that drives rumen microbiota dysbiosis by mediating the host immune function. In the present study, we combined high-throughput 16S rRNA gene sequencing, metagenomics, and immune factor analysis to more deeply explore the mechanisms underlying the response of the rumen microbial community to mastitis, providing a novel perspective on the complex etiology and treatment of mastitis and improving dairy cow health through nutritional strategies.

## RESULTS

### Differences in milk quality and serum parameters between H-SCC and L-SCC dairy cows

**Fig 1:**
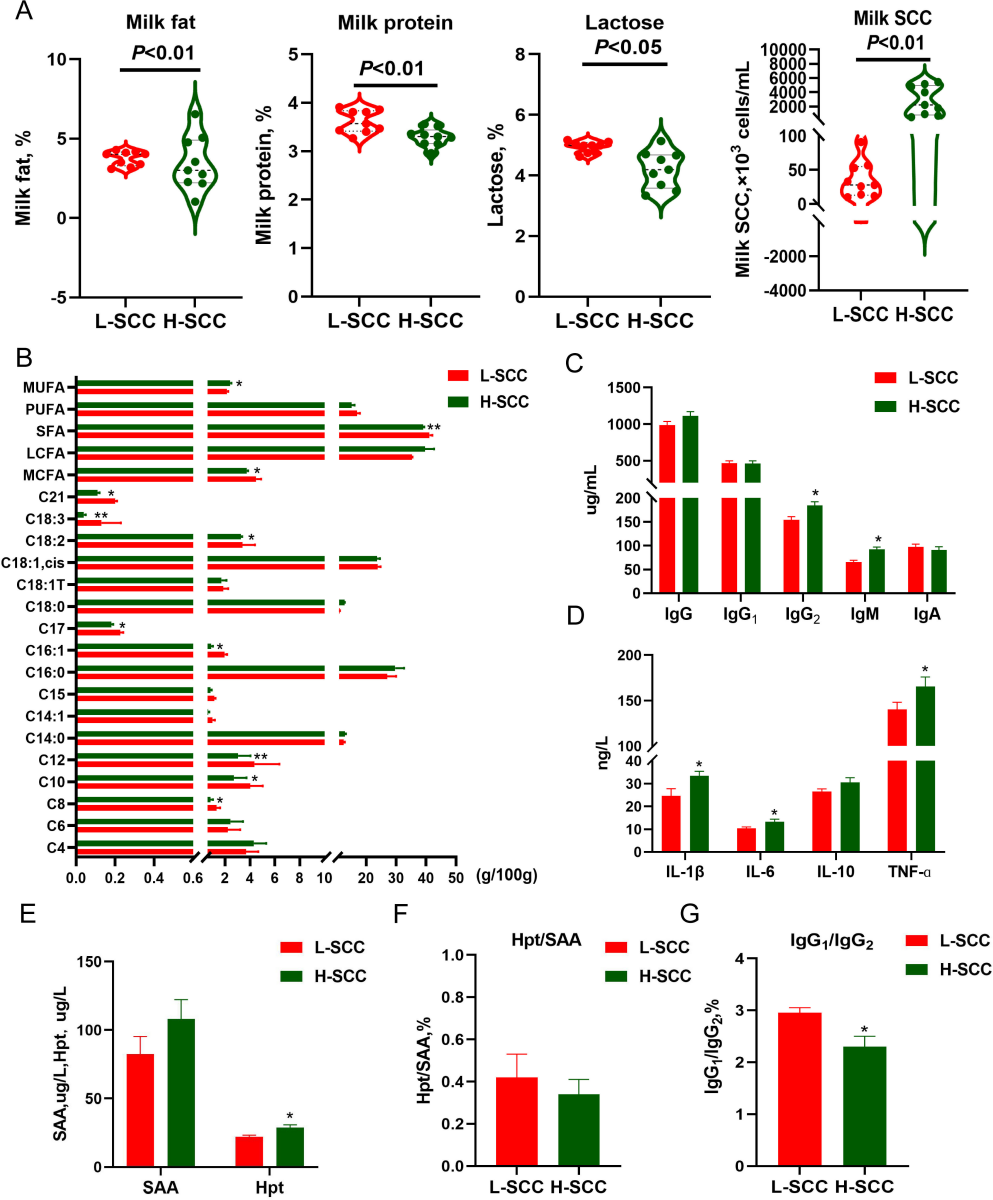
Differences in milk composition and serum parameters between L-SCC and H-SCC dairy cows. (**A**) Milk composition (milk fat, %; milk protein, %; lactose, %; milk SCC, ×10^3^ cells/mL). (**B**) Fatty acid content in the milk of L-SCC cows compared with H-SCC cows. (**C**) Serum immunoglobulin content. (**D and E**) Serum cytokine content. (**F**) Hpt/SAA ratio. (**G)** IgG1/IgG2 ratio.

To investigate the health and milk quality of dairy cows with different SSCs, we analyzed milk composition, immunoglobulin (Ig) levels, and inflammatory markers. The results showed that the fat (*P* < 0.01), protein (*P* < 0.01), and lactose (*P* < 0.05) contents of the milk were significantly lower in the H-SCC group than in the L-SCC group (Fig. 1A). In terms of fatty acid characteristics, the levels of caprylic acid (C8:0), capric acid (C10:0), palmitoleic acid (C16:1, cis-9), heptadecanoic acid (C17:0), octadecadienoic acid (C18:2, cis-9,12), and icosasaccharides (C21:0), and the levels of lauric acid (C12:0) and linolenic acid (C18:3, cis-9,12,15) were significantly lower (*P* < 0.01) in the H-SCC group compared with the L-SCC group (Fig. 1B). Also, the percentages of medium-chain fatty acids, MCFAs, (*P* < 0.05) and saturated fatty acids, SFAs, (*P* < 0.01) were lower in the H-SCC group than in the L-SCC group. No significant differences were found between the groups for polyunsaturated fatty acids (PUFAs) or long-chain fatty acids (LCFAs).

The levels of immunoglobulin G2 (IgG2) and immunoglobulin M (IgM) in the H-SCC group were significantly greater (*P* < 0.05) than those in the L-SCC group, whereas there was no significant difference in the content of immunoglobulin A (IgA) (Fig. 1C). The levels of interleukin-1β (IL-1β), interleukin-6 (IL-6), tumor necrosis factor-ɑ (TNF-ɑ), and haptoglobin (Hpt) were significantly greater (*P* < 0.05) in the H-SCC group than in the L-SCC group, but there were no significant differences in interleukin-10 (IL-10) or amyloid A (SAA) (Fig. 1D and 1E). Moreover, the IgG1/IgG2 ratio was lower in the H-SCC group than in the L-SCC group (*P* < 0.05), as shown in Fig. 1G.

### Rumen interface properties and fermentation parameters

**Fig 2:**
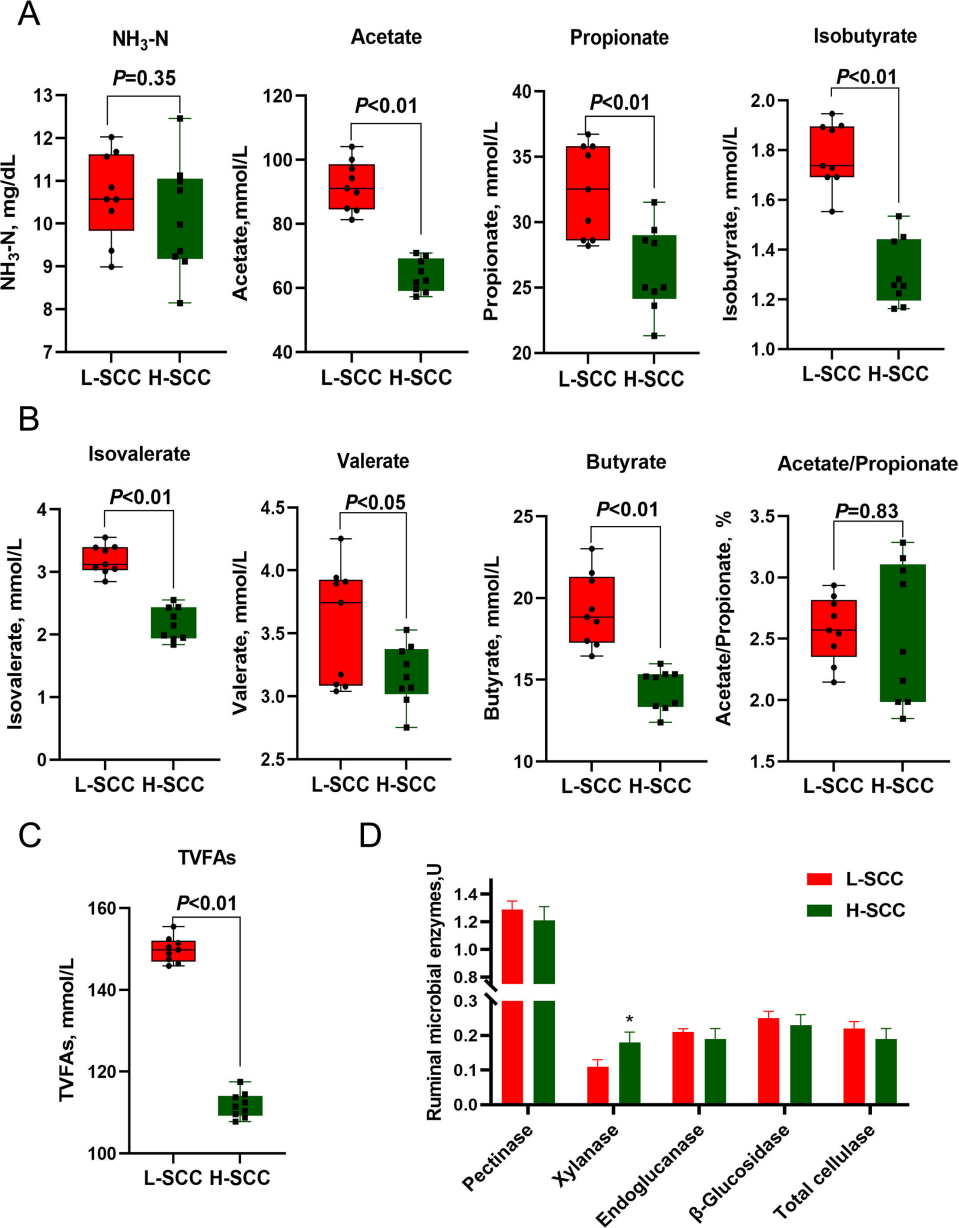
Rumen interface characteristics and fermentation parameters of L-SCC and H-SCC dairy cows. (**A**) Rumen fermentation indicators (NH_3_-N, acetate, propionate, and isobutyrate). (**B**) Rumen fermentation indicators (butyrate, isovalerate, valerate, and A/P ratio). (**C**) Rumen total volatile fatty acids (TVFAs). (**D**) Rumen microbial enzymes (pectinase, xylanase, endoglucanase, β-glucosidase, and total cellulase).

To determine the detailed rumen interface properties and fermentation parameters, we measured the pH, surface tension, redox potential, surface hydrophobicity, rumen microbial enzymes, ammonia nitrogen, and total volatile fatty acids (TVFAs) in the L-SCC group and the H-SCC group. As shown in Table 1, the redox potential was higher (*P* < 0.05) in the H-SCC group than in the L-SCC group. In contrast, the surface tension and cell surface hydrophobicity were significantly lower (*P* < 0.05) in the H-SCC group, but there were no statistically significant differences in pH (Table 1) or ammonia nitrogen content between the two groups (Fig. 2A). Compared with the L-SCC group, the total volatile acid content in the H-SCC group was significantly decreased (*P* < 0.05), along with the valeric acid content (*P* < 0.05) and the acetic acid, propionic acid, isobutyric acid, butyric acid, and isovaleric acid contents (*P* < 0.01) (Fig. 2A to C). Xylanase activity was dramatically higher in the H-SCC group than in the L-SCC group (*P* < 0.05), but no differences were detected in the other rumen microbial enzymes (Fig. 2D).

**TABLE 1:** Physicochemical properties of the dairy cow rumen interface

Property	L-SCC	H-SCC	*P* value
pH	6.49 ± 0.06	6.27 ± 0.07	0.099
Surface tension (N/m)	56.57 ± 2.73	49.04 ± 1.12	0.014
Redox potential (mV)	−295.43 ± 15.01	−102.125 ± 15.72	0.001
Surface hydrophobicity	0.19 ± 0.01	0.16 ± 0.01	0.021

### Response of rumen microbial composition profiles to different somatic cell counts

**Fig 3:**
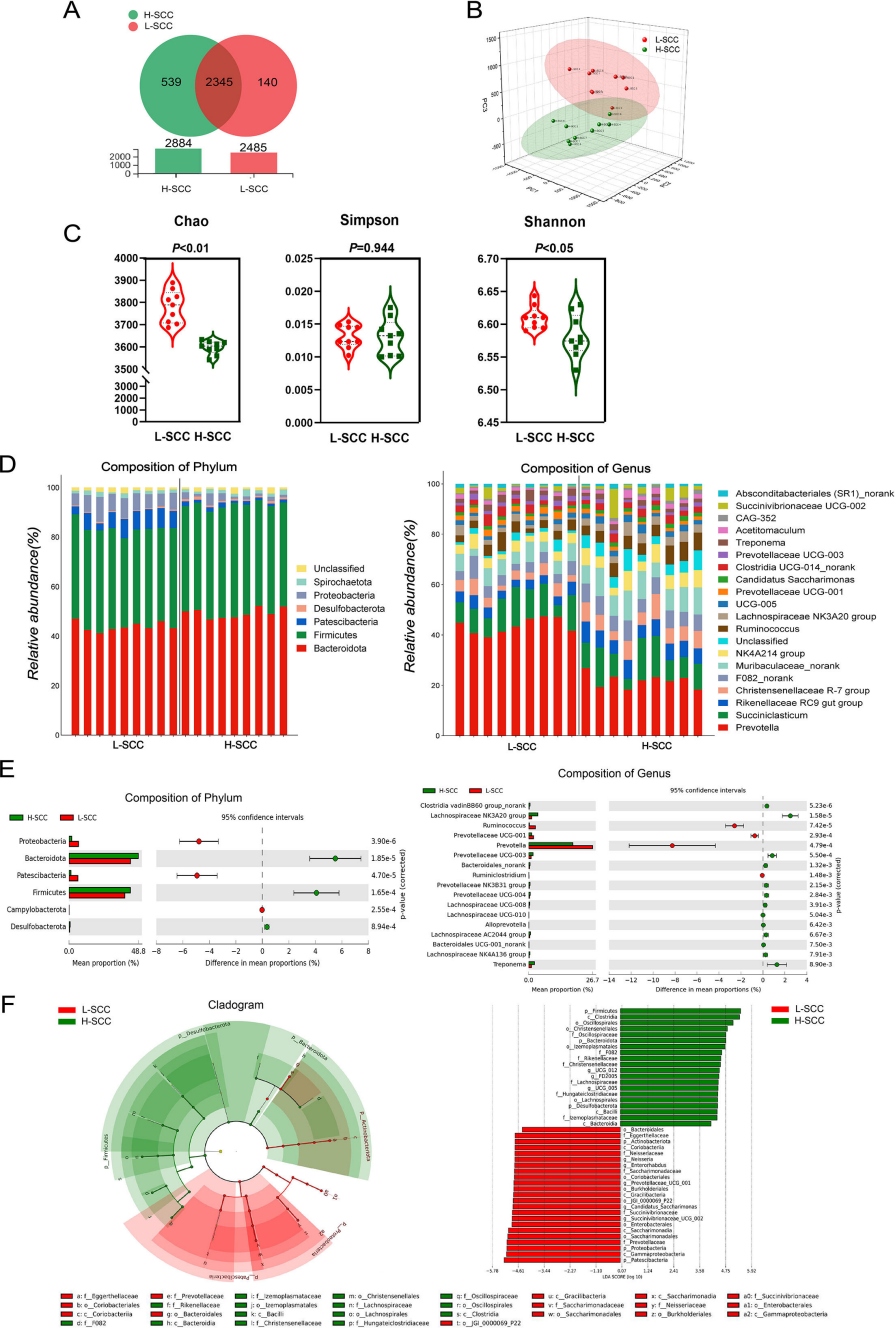
Analysis of differences in rumen microbial composition. (**A**) OTU Venn analysis. (**B**) Multi-sample PCA. (**C**) Alpha diversity analysis (Shannon, Chao, and Simpson). (**D**) Bacterial community composition at the phylum and genus levels. (**E**) Differential abundance of rumen flora at the phylum and genus levels. (**F**) LEfSe analysis was performed to determine the different bacterial taxa enriched in the L-SCC and H-SCC groups (lg LDA score > 3.5).

To determine the potential relationship between rumen microbes and high and low SCCs, we employed high-throughput 16S rRNA gene sequencing. In this study, 722,319 high-quality sequences were obtained from 18 samples, with an average length of 417.8 bp per sequence. We clustered the high-quality sequences with ≥97% sequence identity, resulting in 5369 operational taxonomic units (OTUs; Fig. 3A). For the beta-diversity analysis, the PCA results clearly showed clustering between the H-SCC group and the L-SCC group (Fig. 3B). As shown in Fig. 3C, the alpha diversity indices, Chao (*P* < 0.01) and Shannon (*P* < 0.05), were significantly lower in the H-SCC group than in the L-SCC group. In contrast, no difference was found in the Simpson index (*P* = 0.944). In total, 32 phyla and 414 genera of bacteria were obtained. The dominant bacterial phyla included *Bacteroidota* (48.25% ± 0.58%), *Firmicutes* (36.49% ± 0.32%), *Proteobacteria* (3.49% ± 0.45%), *Patescibacteria* (5.47% ± 0.73%), *Spirochaetota* (3.86% ± 0.27%), and *Desulfobacterota* (2.43% ± 0.49%). Among bacterial families, the *Rikenellaceae* (4.75% ± 1.42%) were modestly represented. The dominant genera included *Prevotella* (29.75% ± 3.48%) and *Succiniclasticum* (6.98% ± 1.45%) (Fig. 3D).

Microbial profiles from high-throughput 16S rRNA gene sequencing for differential abundance comparison at the phylum level, for H-SCC compared with L-SCC, the abundance of *Bacteroidota* (*P* < 0.01), *Firmicutes* (*P* < 0.01), and *Desulfobacterota* (*P* < 0.01) were significantly greater, whereas the abundance of *Proteobacteria* (*P* < 0.01) and *Patescibacteria* (*P* < 0.01) was significantly lower in the H-SCC group. Among orders and families, the *Prevotellaceae* UCG-001 (*P* < 0.01) was significantly greater in the L-SCC group than in the H-SCC group, whereas the abundance of *Lachnospiraceae* (*P* < 0.01) and Bacteroidales (*P* < 0.01) were significantly lower in the L-SCC group than in the H-SCC group (Fig. 3E). At the genus level, the abundance of *Ruminococcus* (*P* < 0.05) and *Prevotella* (*P* < 0.01) were significantly higher in the L-SCC group than in the H-SCC group, and *Clostridia* (*P* < 0.01) was lower.

The nonparametric factorial Kruskal‒Wallis rank sum test was used to conduct a linear discriminant effect size (Lefse) analysis of the rumen bacteria between the two groups. There were 43 differential bacterial species between the two groups. Of these, 20 species were more abundant in the H-SCC group than in the L-SCC group (LDA > 3.5, *P* < 0.05), and 23 species were significantly enriched in the L-SCC group (LDA > 3.5, *P* < 0.05) (Fig. 3F).

### Rumen microbial functions as determined by metagenomics

**Fig 4:**
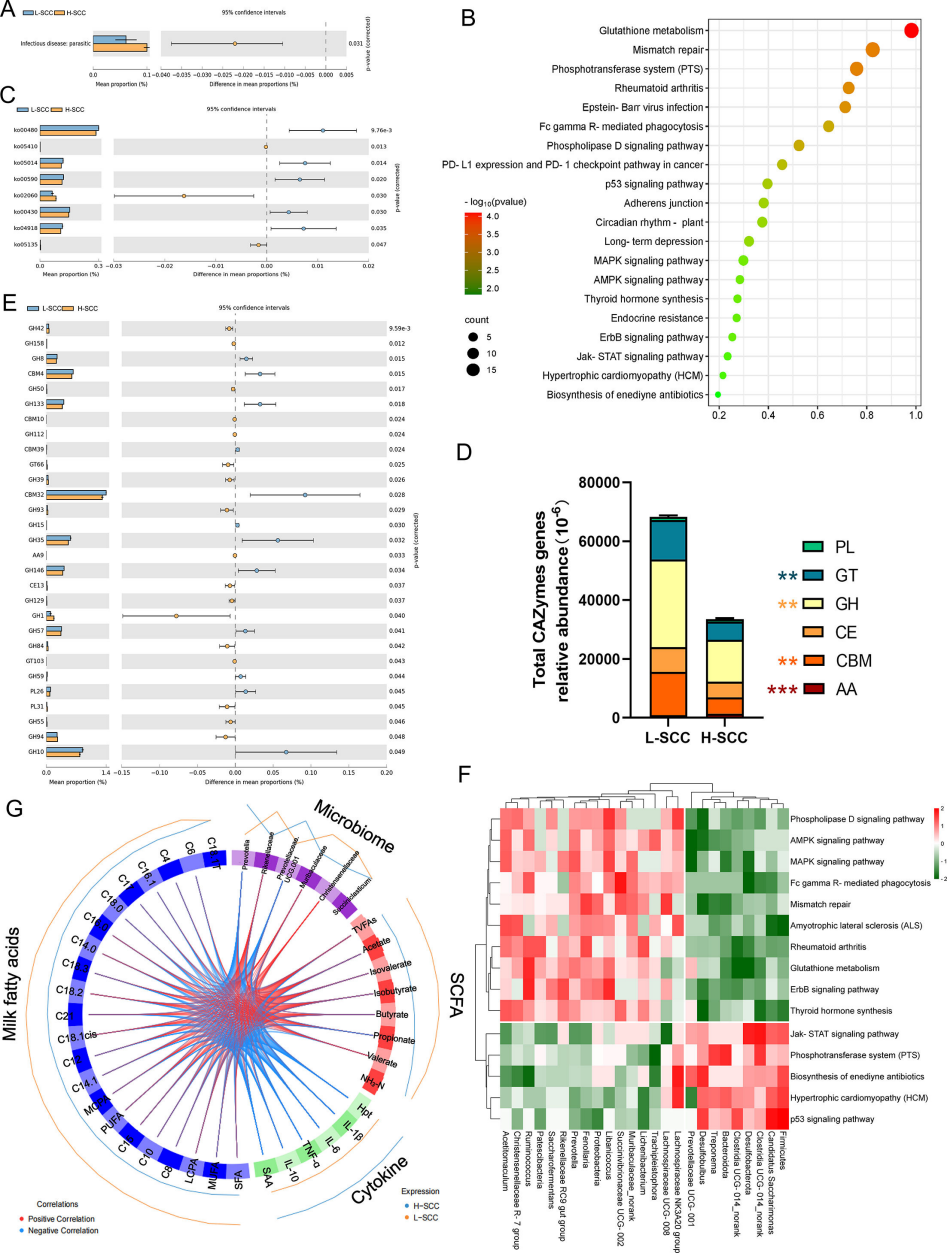
Analysis of the correlations among rumen microbial function, rumen fermentation parameters, and lactation performance. (**A**) KEGG functions at level 2 were tested by the Wilcoxon rank-sum test. (**B**) KEGG functions at level 3 were tested by the Wilcoxon rank-sum test. Axis: rumen microorganisms; points: abundance. The color of the points indicates *P* values (blue: 0.01 < *P* < .05, red: *P* < 0.01). (**C**) Differential pathways within the enriched KEGG ontologies. (**D**) Abundance of carbohydrate-active enzymes in dairy cows. GH, glycoside hydrolase; GT, glycosyl transferase; PL, polysaccharide lyase; CE, carbohydrate esterase; AA, auxiliary activity; CBM, carbohydrate-binding module. (**E**) Abundance analysis of carbohydrate enzyme genes encoded by dairy cow rumen microorganisms. (**F**) Heatmap showing correlation patterns of associations between differential microbial species and metabolic pathways. Correlation analyses were conducted using Spearman’s rank correlation. Only strong (correlation coefficient [*R*] > 0.5 or < −0.5) and significant (*P* < 0.05) correlations were selected for display. (**G**) Positive and negative correlations (*r* > 0.6) are indicated by red and blue links, respectively. The combined analysis of rumen microorganisms, serum cytokines, rumen total volatile fatty acids (TVFAs), and milk fatty acids was performed by DIABLO (data integration analysis for biomarker discovery using a latent component method for omics).

The metagenomic binning method was employed for KEGG enrichment analysis using metagenomic data to investigate the functional differences between L-SCC and H-SCC cows ( Fig. S1 to S3). We selected the level-2 vital pathways, and 46 categories were obtained. Remarkably, one of these pathways was related to infectious disease and parasitic disease (FDR-adjusted *P* < 0.05), which was significantly enriched in the H-SCC group (Fig. 4A). The metagenomic sequences were mapped to 399 category-3 pathways. KEGG pathway enrichment analysis by DAVID revealed that the top 20 pathways in which the differentially expressed proteins were enriched were glutathione metabolism, the phosphotransferase system (PTS), Epstein‒Barr virus infection, the MAPK signaling pathway, the phospholipase D signaling pathway, thyroid hormone synthesis, hypertrophic cardiomyopathy (HCM), the ErbB signaling pathway, long-term depression, the P53 signaling pathway, and the Jak-STAT signaling pathway (FDR-adjusted *P* < 0.05) (Fig. 4B). We further analyzed significant differences at the third level for all the KO pathways annotated by each module. As shown in Fig. 4C, there were significant differences (*P* < 0.05) between the two groups in the ko00480 pathway (glutathione metabolism), ko05014 pathway (amyotrophic lateral sclerosis), ko00590 pathway (arachidonic acid metabolism), ko00430 pathway (taurine and hypotaurine metabolism), and ko04918 pathway (thyroid hormone synthesis). However, the ko05410 pathway (hypertrophic cardiomyopathy), ko02060 pathway (phosphotransferase system), and ko05135 pathway (Jebuzanian infection) were significantly increased in the H-SCC group compared with the L-SCC group (*P* < 0.05).

To better understand the mechanism by which the rumen microbiota induced changes in milk SCC, CAZymes analysis was performed. As shown in Fig. 4D, glycoside hydrolase (GH) accounted for the greatest abundance among the CAZymes; this was followed by glycosyltransferase (GT). Thus, the CAZymes were significantly enriched in GH, GT, carbohydrate esterase (CE), carbohydrate-binding module (CBM), polymer lyase (PL), and auxiliary activities (AA). The abundance of carbohydrate enzyme-encoding genes in rumen microorganisms in the two groups was determined, and 29 CAZyme genes with differences between groups were identified (Fig. 4E). Among them, 19 genes encoded structurally related glycoside hydrolases, and eight of these genes, GH8, GH133, GH15, GH35, GH146, GH57, GH59, and GH10, were significantly reduced in abundance in the H-SCC group (*P* < 0.05). The abundance of the remaining GH42, GH158, GH50, GH122, GH39, GH93, GH129, GH1, GH84, GH55, and GH94 genes was significantly greater in the H-SCC group than in the L-SCC group (*P* < 0.05).

Relationships between differentially abundant microbial taxa and functions were constructed using Spearman correlation data. A total of 25 taxa showed significant relationships with 15 differentially abundant pathways (*R* > 0.50 and *P* < 0.05, Fig. 4F). The abundance of 20 genera, including *Ruminococcus*, *Libanicoccus*, *Prevotella*, *Fenollaria*, and *Lichenibacterium*, were positively correlated with the glutathione metabolism pathway (*R* > 0.50, *P* < 0.05), and the *Firmicutes*, *Bacteroidota*, *Clostridia UCG-014_norank*, *Treponema*, *Desulfobulbus*, *Candidatus, Saccharimonas*, etc., were positively correlated with the phosphotransferase system (PTS) pathway. In particular, the *Prevotellaceae UCG-001* was positively correlated with hypertrophic cardiomyopathy (HCM) and the Jak-STAT signaling pathway but negatively associated with glutathione metabolism, the AMPK signaling pathway, and Fc gamma R-mediated phagocytosis. In addition, data integration analysis for biomarker discovery using a latent component method for omics (DIABLO) was employed to investigate the correlations among rumen microorganisms, serum cytokines, rumen TVFAs, and milk fatty acids (Fig. 4G). The families (*Rikenellaceae*, *Muribaculaceae*, and *Christensellaceae*) were positively correlated with fatty acids from ruminal fermentation, such as valerate, butyrate, and TVFAs, and negatively correlated with cytokines. Milk fatty acids (C6, C4, C16, C17, and C18.0), and cytokines were positively associated with *Prevotella* and *Prevotellaceae* UCG-001. Milk fatty acids in (C12, C14, C14.1, C18.2, and C21) were positively correlated with *Rikenellaceae*, *Muribaculaceae,* and *Christensellaceae*.

### Rumen microbial interactions and functional pathways identified in co‐occurrence networks

**Fig 5:**
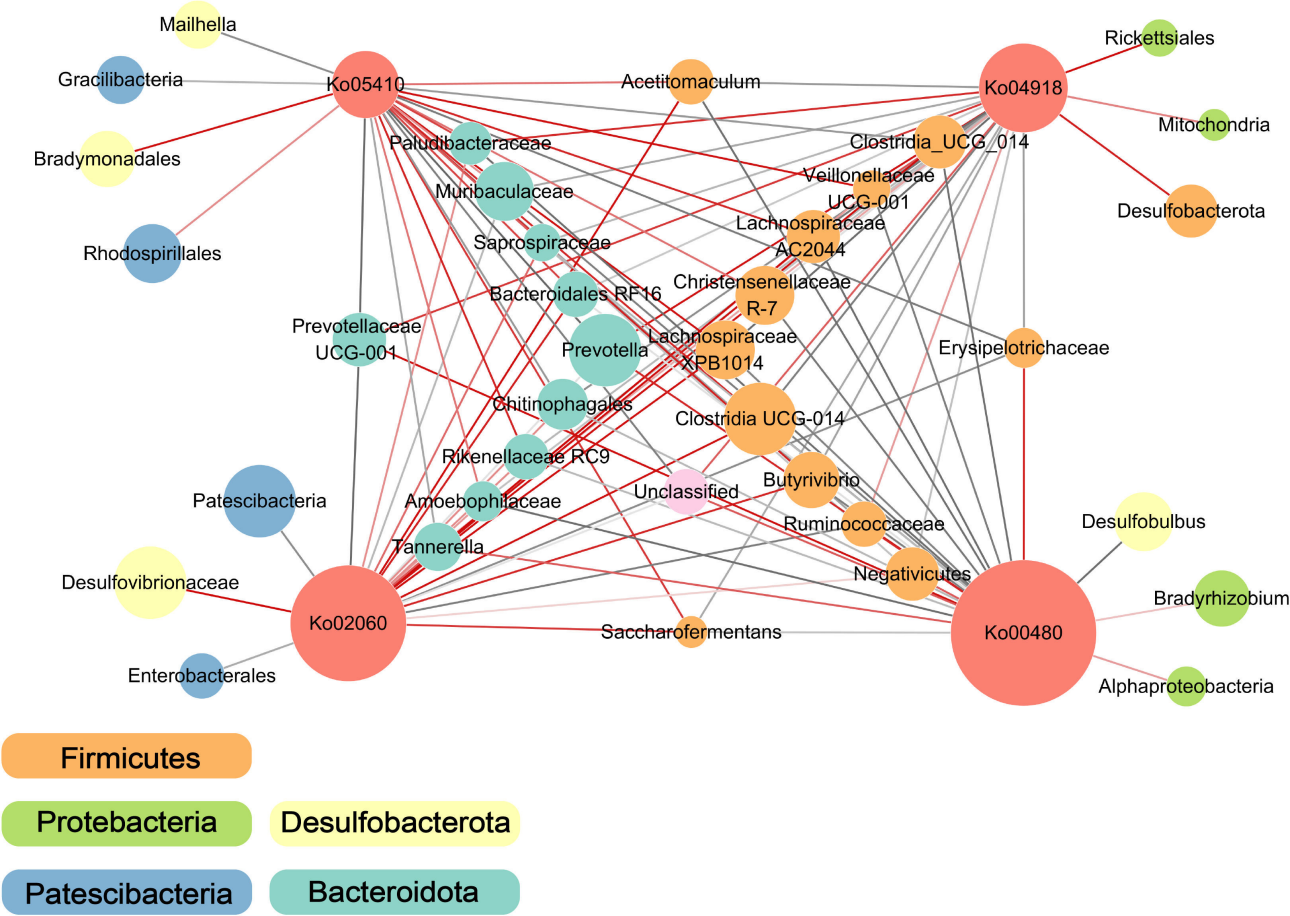
Co-occurrence networks of the rumen bacteria and associated microbial functions in the L-SCC and H-SCC groups. The blue edges indicate positive relationships, and the red edges indicate negative relationships. The node size is proportional to the mean abundance. Ko05410, hypertrophic cardiomyopathy (HCM); Ko04918, thyroid hormone synthesis; Ko02060, phosphotransferase system (PTS); Ko00480, glutathione metabolism .

We performed a co-occurrence network analysis, which revealed 228 co-occurrence relationships of rumen microbiota interactions with enriched functional pathways between the H-SCC and L-SCC groups. In the rumen microbiome response to different SCCs, 186 connections were found. The most positive relationships occurred among the *Firmicutes*, and the most antagonistic relationships occurred for *Bacteroidetes* among the ko05014 pathway (amyotrophic lateral sclerosis), ko04918 pathway (thyroid hormone synthesis), ko02060 pathway (phosphotransferase system), and ko00480 pathway (glutathione metabolism) (Fig. 5). Notably, ko04918 and ko00480, which were significantly enriched, had positive relationships with *Proteobacteria*. The most negative correlation was observed between the ko00480 pathway and *Firmicutes*, whereas the ko02060 pathway was positively correlated with *Bacteroidota*. Interestingly, *Prevotella*, *Muribaculaceae*, and *Prevotellaceae* were the major bacteria of *Bacteroidota*, and a larger node represents higher abundance. The *Clostridia*, *Lachnospiraceae*, and *Christensenellaceae* were the dominant bacteria in the *Firmicutes*.

## DISCUSSION

Mastitis is a severe disease that reduces milk production and quality, causing significant annual economic losses (21). The SCCs are an indicator of the health of the mammary gland, and this diagnostic parameter for mastitis plays an important role in the milk industry. In this study, the rumen microbiota and enriched functional pathways of dairy cows with high or low SCCs were related through their significant influences on ruminal fermentation characteristics, carbohydrate-active enzyme activities, and the levels of serum cytokines. We demonstrated that rumen fermentation metabolites and microbiota play a central role in the host’s regulation of immune function and enrichment in functional pathways, including glutathione metabolism, the phosphotransferase system (PTS), and thyroid hormone synthesis. Similarly, research has shown that rumen bacteria and their metabolites can migrate to distal extraintestinal organs, such as mammary glands. It is speculated that the occurrence of mastitis is related not only to infection of the mammary gland by external pathogenic microorganisms but also to gastrointestinal pathogenesis (22). Thus, this study analyzed the correlation between the structure and function of the dairy cow rumen microbiota and high and low SCCs, which should provide a better understanding of the pathogenesis of mastitis in response to gastrointestinal dysbiosis.

Mounting evidence has shown an important role for different SCCs in milk quality and the host’s immune response. As expected, we found that the contents of milk fat and lactose and the milk fatty acid yield were significantly decreased in the H-SCC group. In particular, the concentration of caprylic acid (C8:0), capric acid (C10:0), palmitoleic acid (C16:1, cis-9), heptadecanoic acid (C17:0), and octadecadienoic acid (C18:2, cis-9, 12), and other milk fatty acids decreased in correspondence to high SCCs. Wang et al. (23) showed that LPS-induced mastitis could significantly reduce milk fat synthesis. In addition, the impaired blood-milk barrier in high-SCC cows led to the downregulation of lactose synthase activity, resulting in a significant decrease in lactose concentration in the blood (24). It is well known that the content of natural antibodies (NAbs) in blood and milk samples is highly important for indicating the health status of cows (25). When inflammation occurs, immune recognition is mainly achieved by IgG2 and IgM (26). In the present study, the levels of IgM and IgG2 were greater in the H-SCC group than in the L-SCC group, demonstrating that the host was in a state of inflammation, which is also consistent with the findings of Li et al. (27). However, when mastitis occurs in dairy cows, TNF-α and IL-1β are rapidly expressed in the early stages of infection and have powerful proinflammatory functions. Moreover, serum amyloid A (SAA) and haptoglobin (Hpt) are important markers for the determination of bovine mastitis (28, 29). Our results indicate that the levels of IL-1β, IL-6, Hpt, and (TNF-α) in the H-SCC group increased significantly, in agreement with the findings of Guo et al. (30). Although evidence indicates that different SCCs are associated with host immune status, whether ruminal dysbiosis is caused by inflammation or milk somatic cell migration to the rumen remains unknown.

Differences in feed intake between cows with low and high SCCs should always be considered because feed intake significantly affects ruminal fermentation, nutrient availability, and milk composition. Cows with high SCC often have subclinical or clinical mastitis and also tend to reduce their feed intake because of systemic effects such as inflammation, pain, and discomfort. This reduction alters ruminal fermentation, leading to decreased volatile fatty acid (VFA) production, shifts in microbial populations, and reduced nutrient utilization efficiency. In our previous study, the high-yield (HY) group exhibited significantly greater dry matter intake (DMI), milk production, and component yields compared with the low-yield (LY) group, with no significant difference in SCC observed between the groups (31). Research by Xue et al. (19, 20) concluded that high milk protein yield (MPY) in dairy cows was not only influenced by feed, management, age, and lactation stage but also closely associated with differences in the rumen microbiome, its metabolites, and their utilization by the host. The rumen and serum metabolome data collectively explained over 95% of the variation in MPY. These findings underscore the critical role of rumen microbes in SCC regulation, suggesting that a deeper understanding of microbial composition and metabolic interactions may provide valuable insights for improving dairy cow health and productivity.

The rumen microbes exert their effects on host regulation through changes in the levels of specific metabolites. Rumen-fermented metabolites such as VFAs can act as energy sources and precursors to milk components. Wang et al. (32) reported that with the development of mastitis, the ruminal lactic acid, acetic acid, propionic acid, butyric acid, valeric acid, and total VFA concentrations decreased significantly, which was in line with our findings that these concentrations were significantly lower in the H-SCC group compared with the L-SCC group. The role of VFA as a precursor for milk components (33) could account for some of the changes in the milk. A previous study showed that the rumen levels of lactic acid and TVFAs were significantly reduced in cows with mastitis progression (34). Other studies have shown that ruminal fluid surface tension was positively correlated with NH_3_-N, feed digestibility, and microbial protein production (35). When the redox potential was increased, the oxygen concentration in the rumen was elevated, which is not conducive to the survival of beneficial bacteria in the rumen (36). This confirms that dysbiosis of rumen microbial populations in mastitis cows is positively correlated with an increase in redox potential, which is in line with our results. Thus, the changes in these rumen fermentation parameters can be correlated with changes in the rumen environment of dairy cows with different SCCs. This finding indicates a novel selective opportunity for improving overall health by regulating rumen microbial metabolism.

This study revealed that the rumen microbiome is closely associated with changes in SCCs and has been strongly linked to host metabolite regulation and health status. Characterization of the ruminal microbiota is commonly performed using metagenomic and 16S rRNA gene high-throughput sequencing. The key difference between these approaches is that metagenomic sequencing offers comprehensive insights into microbial functional pathways and genetic potential, whereas high-throughput 16S rRNA gene sequencing primarily focuses on characterizing microbial diversity and taxonomy. In the present study, we employed both metagenomic and 16S rRNA gene sequencing to comprehensively characterize the differences in rumen microbiota between the H-SCC and L-SCC groups. Previous studies have indicated that changes in the rumen microbiota can result in an alteration in fermentation products, thereby triggering the release of LPS endotoxin that induces mastitis (13, 21, 37). Our results revealed that the abundance of *Bacteroidetes* was significantly increased in the H-SCC group. A previous study showed that *Bacteroides* species mainly produce propionic acid and encode carbohydrate-active enzymes that regulate inflammatory factors and break down polysaccharides (38). This is associated with changes between the H-SCC and L-SCC groups, in which inflammatory cytokines, such as IL-1β and TNF-α, were significantly increased in the H-SCC group. Some important ruminal cellulolytic species, such as *Ruminococcus* albicans, *Fibrobacterium* succinate, and *Prevotella*, can also secrete pectin-lysing enzymes (39), which could explain why the TVFAs significantly decreased with decreasing *Prevotella* abundance in the H-SCC group. Xue et al. (40) demonstrated that the abundance of *Prevotella* was significantly negatively correlated with the milk fat rate and was related to the biosynthetic functions of branched-chain amino acids, ruminal amino acids, and serum amino acids, providing sufficient precursor materials for the synthesis of immunoglobulins, which was in agreement with our results. Furthermore, it has been reported that the genera, *Lactococcus*, *Fibrobacterium*, and *Bacteroidetes,* can degrade pectin, and their abundance in the rumen is related to the pectin content in the feed (41). The above evidence shows the functional interdependence of rumen microorganisms, which can efficiently decompose dietary fiber by secreting different carbohydrate-degrading enzymes, such as xylanase. In our study, the H-SCC group had fewer CAZy protein-coding genes. CBM10, which is related to cellulose binding, was significantly increased, and CBM32, which is associated with the binding of galactose and lactose, was significantly reduced in the H-SCC group. This is consistent with the study of Liu et al. (42).

*Muribaculaceae* is also an essential microbial population in the rumen that is positively correlated with propionate and butyrate concentrations and longevity and negatively correlated with diarrhea (43, 44). In particular, we identified *Muribaculaceae* as the principal family in the phylum Bacteroidota, which was significantly positively correlated with the phosphotransferase system (PTS) pathway. When the composition and abundance of the rumen microbial population changes, the abundance of genes in each family of CAZymes that affect rumen metabolite production changes, making it reasonable to conclude that the rumen microbes can promote the development of mastitis through their responses to metabolites and host immunity.

Metagenomics has revealed the functional genes of the microbiome, offering insights into the potential roles of rumen microbes in health and disease (45). To explore these roles, we performed a metagenomic analysis of the KEGG pathways. The analysis identified genes associated with differences in microbiota between the H-SCC and L-SCC groups, which were primarily linked to the glutathione pathway, the phosphotransferase system, thyroid hormone synthesis, and hypertrophic cardiomyopathy. Previous metagenomic studies of dairy cow rumen samples have suggested that inulin supplementation may influence mastitis via pathways such as amino acid metabolism, protein digestion and absorption, arachidonic acid metabolism, and glycerophospholipid metabolism (46). Although consistent with our findings, these results highlight potential associations rather than direct cause-effect relationships.

Glutathione is known to protect cells from oxidative damage and maintain redox homeostasis (47), which could explain the observed relationship between glutathione metabolic pathways and oxidative stress in mastitis (48). Moreover, earlier research indicates that the regulatory effects of antioxidant stress drugs on glutathione metabolism were associated with changes in the abundances of *Prevotella* and *Escherichia* (49). This could explain the connection between the lower abundance of *Prevotella* and the differences in bacterial enrichment in the glutathione metabolism pathway observed in the H-SCC group. The taurine and hypotaurine metabolic pathways are also related to oxidative stress. Taurine activates the Keap1-Nrf2-ARE signaling pathway, enhancing antioxidant gene expression (50). Thomas et al. (51) found elevated levels of arachidonic acid and other metabolites, along with inflammatory mediators, in cows with streptococcal mastitis.

To better understand these relationships, we examined correlations between microbial species and microbial functions. Our results indicated that bacteria such as *Clostridia*, *Lachnospiraceae*, and *Christensenellaceae* were negatively correlated with the glutathione metabolism pathway. The Circos plot revealed positive correlations between *Rikenellaceae*, *Muribaculaceae*, *Christensenellaceae,* and fatty acids such as valerate, butyrate, and TVFAs produced by ruminal fermentation, as well as negative correlations with cytokines. Tavella et al. (52) also associated *Christensenellaceae*, *Porphyromonadaceae*, and *Rikenellaceae* with better metabolic health. These findings suggest that beneficial bacteria in the rumen microbiota, such as *Rikenellaceae*, *Muribaculaceae*, and *Christensenellaceae*, may increase rumen FA content through metabolic pathways such as glutathione metabolism and the phosphotransferase system, potentially increasing the fatty acids and cytokines in milk and contributing to improved host health. These observed correlations should not be interpreted as direct causal relationships but as insights into complex microbial-host interactions requiring further investigation.

### Conclusions

**Fig 6:**
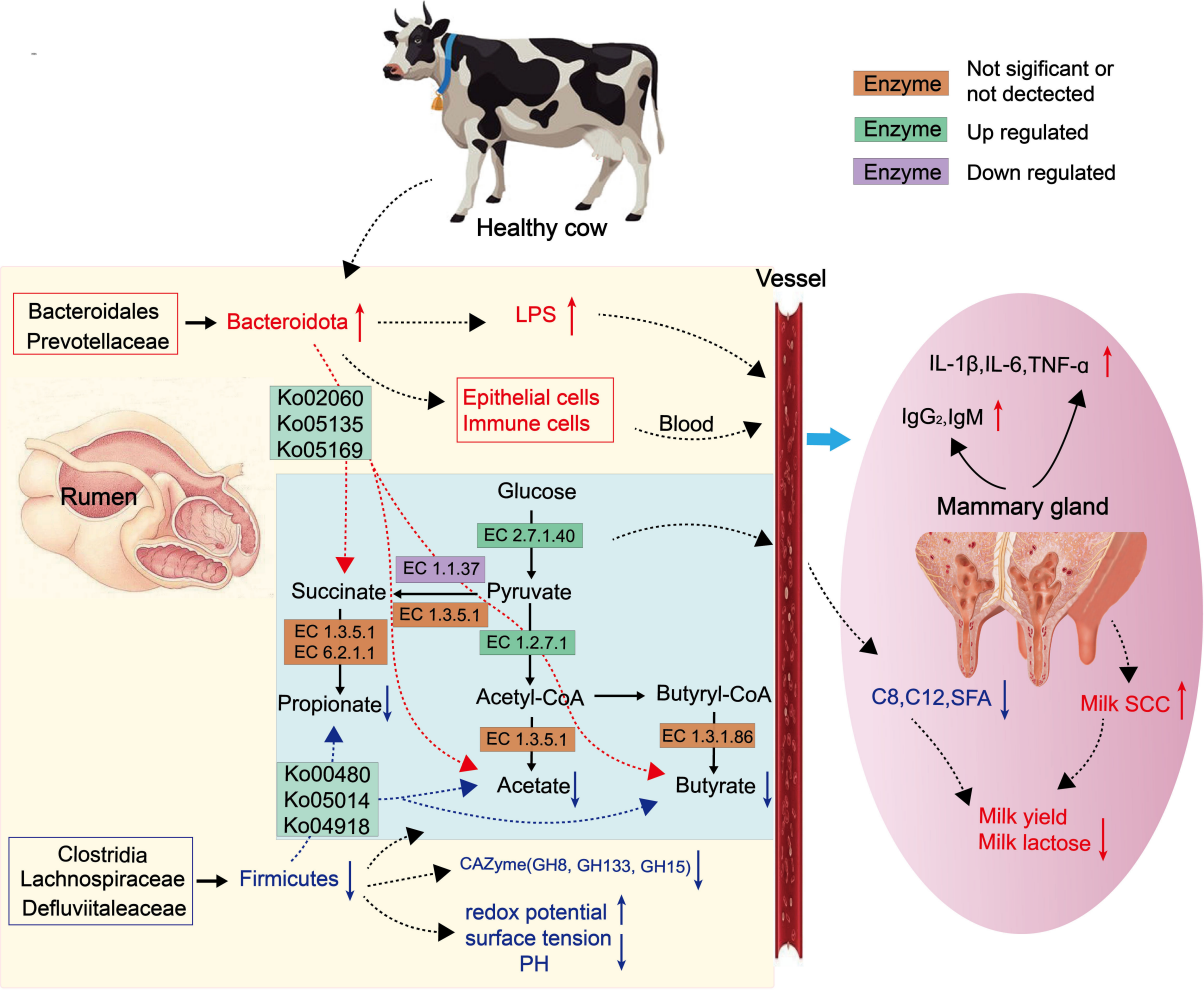
A working model to illustrate the main findings by integrating all the rumen microbiota, fermentation parameters, and serum cytokines associated with different somatic cell counts in dairy cows. LPS, lipopolysaccharide; SCCs, somatic cell counts; C8, C12, volatile fatty acids; CAZyme, carbohydrate active enzymes; IL-1β, interleukin-1β; IL-6, interleukin-6; TNF-α, tumor necrosis factor-α; SFA, saturated fatty acid; IgG_2_, recombinant immunoglobulin G2; IgM, recombinant immunoglobulin M.

This study identified differences in the composition of rumen microbiota and fermentation metabolites between the H-SCC and L-SCC groups. By focusing on the types and abundance of taxa in the rumen microbiota, we found that differentially regulated pathways, such as glutathione metabolism, thyroid hormone synthesis, and the phosphotransferase system, collectively can explain the role of ruminal microorganism function in the mastitis-related changes in SCC (Fig. 6). We showed that the levels of inflammatory cytokines were significantly higher, and the TVFAs significantly lower in the H-SCC group. These changes were accompanied by a decrease in the abundance of *Prevotella* in the H-SCC group. Regulatory strategies targeting the glutathione metabolic activity of rumen microbiota could provide a novel opportunity for alleviating host dysbiosis and elevated SCCs and improving overall health. Future investigations need to focus on modifications to the ruminal microbes and metabolites and alterations to SCCs caused by various feeding regimens, seasonal effects, and dietary supplements. Likewise, in addition to highlighting the role of the rumen microbiota in mammary gland health, our findings could provide a framework for nutrition strategies to improve immunity and performance in the dairy cow industry.

## MATERIALS AND METHODS

### Animals and experimental design

Eighteen Holstein cows with similar lactation days, parity, and weight were used in this study. According to the number of SCCs in the milk, the cows were divided into a low SCC group (L-SCC, 0–100,000 SCC/mL, *n* = 9) and a high SCC group (H-SCC, >500,000 SCC/mL, but without apparent signs of inflammation in the udder, including udder swelling; *n* = 9) groups. All cows were fed three times a day, at 0700, 1300, and 1900 h. Feed intake was monitored using automated troughs equipped with a Roughage Intake Control System (Marknesse, The Netherlands). Each feeding station featured an individual recognition system that permitted only one cow to access a specific feeding bank at a time, automatically recording its feeding behavior. The ingredients and nutritional levels of the total mixed ration (TMR) containing a 40:60 forage-to-concentrate ratio are shown in Table 2. The fat, protein, lactose, urea nitrogen, and SCC of the milk samples were measured with a Milkoscan 6000 FT (Foss Electric, Hillerød, Denmark).

**TABLE 2:** Composition and nutrient levels of the basal diet (DM basis)

Ingredient	Content (% of DM)^a^
Alfalfa hay	6.9
Corn silage	46.32
Oat grass	2.4
Ground corn	9.88
Soybean meal	5.10
Steam-flaked corn	4.40
DDGS^b^	4.40
Corn bran	3.70
Extruded soybean	3.00
Barley	2.66
Wheat bran	2.66
Sodium cyclamate	2.40
Oats	1.50
Canola meal	1.07
Cottonseed meal	1.07
MAGALAC^c^	0.90
NaHCO_3_	0.59
Limestone	0.48
NaCl	0.27
Premix^d^	0.30
CP	17.40
NDF	31.10
ADF	16.6
Ether extract	5.00
Ca	0.78
P	0.44
NE_L_, Mcal/kg	1.76

^a^ Chemical composition based on chemical analysis of the total mixed ration (TMR), as described.

^b^ DDGS, dried distillers grain with solubles.

^c^ Church and Dwight Co., Inc., Princeton, NJ.

^d^ Formulated to provide (per kg of DM) 4,560 mg of Cu, 3,000 mg of Fe, 12,100 mg of Zn, 4,590 mg of Mn, 60 mg of Co, 200 mg of Se, 270 mg of I, 10,000 IU of vitamin E, 450,000 IU of vitamin D, 2,000,000 IU of vitamin A, and 3,000 mg of nicotinic acid.

### Fatty acid composition of milk

Fatty acid methyl esters (FAMEs) in milk were measured as described by Yuchao et al. (53).

Briefly, 2 mL milk samples were mixed with n-hexane/isopropyl alcohol, Na₂SO₄, and sodium hydroxide/methanol solutions, stirred for 2 min at RT, and then centrifuged at 1,900 × *g* for 20 min at 4°C. The supernatants were collected and dried under nitrogen gas. After adding n-hexane, sodium hydroxide-methanol, and methanol solutions, the mixture was incubated at 50°C for 15 min, then at 90°C for 1.5 h with 10% hydrochloric acid in methanol. After heating, distilled water and n-hexane were added, and the samples were centrifuged as before. The solution was diluted to 10 mL with n-hexane, and 2 g of anhydrous Na_2_S0_4_ was added. A 1.5 mL aliquot was analyzed by gas chromatography (GC-7820A, Agilent) using a flame ionization detector and CP-Sil 88 column. The temperature conditions were 60°C for 10 min, increasing to 200°C at 2°C/min for 30 min, and then to 240°C at 2 °C/min for 19 min. Retention times were compared with standards, and fatty acid concentrations were expressed as g/100 g total fatty acids.

### Serum biochemical parameters

Blood samples were collected from the tail vein of each cow. The blood tubes without additives were left for approximately 2 h at RT to allow clotting before being centrifuged at 3000 × *g*, 4°C, 15 min. Aliquots of serum were stored at −20°C and utilized for measurements of biochemical parameters. Commercially available kits (Beijing Soler Biotechnology Co., Beijing, China) were used to quantitate IL-1β (#H002-1-2), IL-6 (#H007-1-1), IL-10 (#H009-1-2), and TNF-α (#H052-1-2). In addition, the levels of serum amyloid A protein (SAA, #H134), haptoglobin (Hpt, #H136), IgG (#H106-1-1), IgG1 (#H569), IgG2 (#H570), IgA (#H108-1-2), and IgM (#H109-1-1) were determined by ELISA kit (Nanjing Jiancheng Bioengineering Institute, Nanjing, China). All the above biochemical assays were performed according to the manufacturer’s instructions. Absorbance was measured using a microplate reader (Multiskan FC; Thermo Fisher, New York, NY).

### Rumen fermentation

The rumen fluid samples were collected 4 h after the morning feeding via oral stomach tubes following a previous protocol (19). At the end of each rumen fluid collection, the tubes were cleaned thoroughly with warm water, and at the beginning, 50 mL of rumen fluid was discarded to avoid saliva contamination., the mean ruminal pH was measured immediately using a portable pH and redox meter probe (Thermo Scientific Orion A221, Thermo Fisher Scientific, Waltham, MA). Then, all the samples were frozen at −80°C for further analysis of their physicochemical properties, the ruminal interface, microbial enzymes, and microorganisms.

After thawing, the surface tension of the rumen fluid of each group of cows was measured using a fully automatic tensiometer (platinum ring method). Next, the microbial surface hydrophobicity was measured in 2 mL of rumen fluid.

The absorbance at 400 nm (*A*_0_) was measured with a microplate reader; then, the same volume hexadecane was added to each sample tube. After the two phases of the solution had separated, the water phase was removed, and its *A*_400_ was measured (*A*_1_). The cell surface hydrophobicity was calculated as follows: CSH (%) = [(*A*_0_ − *A*_1_)/*A*_0_] × 100.

NH_3_-N concentration in the rumen fluid samples was analyzed spectrophotometrically according to Cao et al. (54). Six milliliters of rumen fluid was mixed with 2.5 mL of phenol chromagen in a centrifuge tube, and 2.0 mL of hypochlorite reagent was added. The sample was placed in a 37°C water bath for 30 min for color development, and the absorbance at 550 nm was measured using a microplate reader was used to measure the ammonia nitrogen content in the rumen fluid. The total volatile fatty acid (TVFA) content was also measured by mixing 5 mL of rumen fluid with 0.15 mL of 25% metaphosphoric acid. The mixture was allowed to stand at room temperature for 30 min, and the TVFA content was measured with a gas chromatograph (GC-7820A, Agilent Technologies, Inc.).

### Determination of rumen microbial population by high-throughput 16S rRNA gene sequencing

Microbial DNA was extracted from the rumen fluid samples using the E.Z.N.A. Stool DNA Kit (Omega Biotek, Norcross, GA, USA) according to the manufacturer’s protocol. The V4-V5 region of the bacterial 16S ribosomal RNA gene (515 F: GTGCCAGCMGCCGCGG; 907 R: CCGTCAATTCMTTTRAGTTT) was amplified by PCR with 3 min initial denaturation at 95°C, followed by 30 cycles of 95°C for 30 s, 55°C for 30 s, and 72°C for 45 s, and a final extension at 72°C for 5 min. The amplicons were purified using the AxyPrep DNA Gel Extraction Kit (Axygen Biosciences, Union City, CA, U.S.) as previously described (10), pooled in equimolar ratios, and subjected to paired-end sequencing on an Illumina-MiSeq platform according to standard procedures.

Microbial sequencing data were processed by quality control with Fastp, sequence merging using FLASH, and operational taxonomic unit (OTU) clustering at 97% similarity with UPARSE (version 7.1, http://drive5.com/uparse/). Chimeric sequences were removed using UCHIME, and taxonomic annotation was performed with the Ribosomal Database Project (RDP) classifier algorithm referencing the SILVA 16S rRNA gene database (http://www.arb-silva.de) at a 70% confidence threshold. This streamlined workflow ensured high-quality data and accurate species identification. Alpha diversity analyses, including richness indices (Chao and Sobs) and diversity indices (Shannon and Simpson), were determined using rarefaction analysis conducted with Mothur software (v.1.21.1).

Beta diversity was assessed using principal component analysis (PCA) based on unweighted UniFrac distances; bacterial taxonomic distributions within sample communities were visualized using the R package. Multivariate analysis of variance (MANOVA) was conducted to further confirm the observed differences. Spearman’s correlation coefficients were assessed to determine the relationship between the microbiota and metabolic factors, such as signaling molecules. The correlation was considered significant when the absolute value of Spearman’s rank correlation coefficient (Spearman’s *r*) was >0.6, and *P* < 0.05 according to the R stats package was considered statistically significant. The R heatmap package and Cytoscape (http://www.cytoscape.org) were used to visualize the relationships through correlation heatmaps and network diagrams, respectively. One-way analysis of variance (ANOVA) was performed to assess the statistically significant differences in diversity indices between samples. Differences were considered significant at *P* < 0.05. Venn diagrams were drawn using the online tool Draw Venn Diagram.

### Metagenomic binning for the analysis of ruminal microbiota

To better understand the microbial population in the rumen, we used the metagenomic binning method of Xie et al. (55). First, total genomic DNA was extracted from the rumen fluid using a Covaris M220 ultrasonicator according to the manufacturer’s instructions. Subsequently, a paired-end library was constructed using a TruSeq DNA prep kit. Bridge PCR was performed using a cBot TruSeq PE cluster kit v3-cBot-HS. Finally, the Truseq SBS kit v3-HS (200 cycles) was used for Illumina NovaSeq 6000 sequencing. Along with the individual assembly of each sample, we also co-assembled the high-quality reads from the same rumen regional samples in each ruminant species using Megahit (v.1.1.1) with the parameters, min-contiglen 500 and -t 40 to recover more assembled contigs. We then used the contigs from both the single-sample assemblies and the co-assemblies (>1.5 kb) independently for metagenomic binning. We used three methods with default parameters (MaxBin, MetaBAT2, and CONCOCT) to bin the sequences based on their configurations and coverage depth. Finally, we integrated the MAGs generated from the different methods using the DAS tool (v.1.1.1). The standard parameters and detailed methods were the same as those previously published (11).

Data analysis first involved optimization processes, such as de-joining, quality shearing, and contamination removal on the original sequence. High-quality sequences were then used for splicing assembly and gene prediction, and the resulting genes were annotated and classified in terms of species and function using the NR, Kyoto Encyclopedia of Genes and Genomes (KEGG), and CAZy databases. The raw image data obtained by Illumina sequencing was converted into sequence data through base calling, and the software Trimomatic (IlluminaClip: adapters.fa:2:30:10 SlidingWindow 4:15 Minlen:75) was used to filter the raw sequencing data. The taxonomic annotation of the sequencing data by species was performed using Kraken2 software. Megahit (https://github.com/voutcn/megahit) was used to assemble metagenomes. CD-hit (http://www.bioinformatics.org/cd-hit/) software was used for clustering data with 95% identity and 90% coverage; the longest gene for each class was used as the representative sequence to construct nonredundant gene sets. Highly accurate sequence alignments for gene abundance estimates were generated from the data using Salmon (https://github.com/COMBINE-lab/salmon). The protein sequences of the predicted genes were compared with those of genes in the NR, eggNOG, KEGG, CARD, CAZy, and other databases to obtain the annotation information of the indicated genes. KofamScan 1.2.0 software was used for KEGG enrichment analysis. CAZy was annotated by the software hmmscan (dbCAN-HMMdb-V8). Based on the above analysis, LEfSe multivariable statistical analysis, difference comparison, and other multidirectional statistical analyses were performed, and the results are visually displayed.

### Statistical analysis

The data shown in the study were obtained from at least three independent experiments, and the experimental data were initially analyzed with Excel 2010. Multiple *t* tests with one per row were performed using GraphPad Prism ver 9.0 (San Diego, CA, USA). Welch’s *t* test was performed on STAMP for functional difference analysis, and the genes were mainly annotated to the KEGG and CAZymes databases. The results are expressed as the mean ± SD. *P* < 0.05 indicated a statistically significant difference, and *P* < 0.01 indicated a very significant difference.

## Data Availability

The raw sequence data were uploaded to the NCBI (PRJNA1103138).

## SUPPLEMENTAL MATERIAL

Supplemental figures msystems.01093-24-s0001.docx Figures S1 to S3.

ASM does not own the copyrights to Supplemental Material that may be linked to, or accessed through, an article. The authors have granted ASM a non-exclusive, world-wide license to publish the Supplemental Material files. Please contact the corresponding author directly for reuse.

## References

[1] Vargas-Bello-Pérez E, Márquez-Hernández RI, Hernández-Castellano LE. 2019. Bioactive peptides from milk: animal determinants and their implications in human health. J Dairy Res 86:136–144. doi:10.1017/S0022029919000384 31156082

[2] Kearney J. 2010. Food consumption trends and drivers. Philos Trans R Soc Lond B Biol Sci 365:2793–2807. doi:10.1098/rstb.2010.0149 20713385 PMC2935122

[3] Paramasivam R, Gopal DR, Dhandapani R, Subbarayalu R, Elangovan MP, Prabhu B, Veerappan V, Nandheeswaran A, Paramasivam S, Muthupandian S. 2023. Is AMR in dairy products a threat to human health? an updated review on the origin, prevention, treatment, and economic impacts of subclinical mastitis. Infect Drug Resist 16:155–178. doi:10.2147/IDR.S384776 36636377 PMC9831082

[4] Gazzola A, Minozzi G, Biffani S, Mattiello S, Bailo G, Piccinini R. 2021. Effect of weeping teats on intramammary infection and somatic cell score in dairy goats. Front Vet Sci 8:622063. doi:10.3389/fvets.2021.622063 34350222 PMC8326401

[5] Hu X, Guo J, Zhao C, Jiang P, Maimai T, Yanyi L, Cao Y, Fu Y, Zhang N. 2020. The gut microbiota contributes to the development of Staphylococcus aureus-induced mastitis in mice. ISME J 14:1897–1910. doi:10.1038/s41396-020-0651-1 32341472 PMC7305118

[6] Hu X, He Z, Zhao C, He Y, Qiu M, Xiang K, Zhang N, Fu Y. 2023. Gut/rumen-mammary gland axis in mastitis: Gut/rumen microbiota-mediated “gastroenterogenic mastitis”. J Adv Res 55:159–171. doi:10.1016/j.jare.2023.02.009 36822391 PMC10770137

[7] Meng M, Li X, Huo R, Ma N, Chang G, Shen X. 2023. A high-concentrate diet induces mitochondrial dysfunction by activating the MAPK signaling pathway in the mammary gland of dairy cows. J Dairy Sci 106:5775–5787. doi:10.3168/jds.2022-22907 37296051

[8] Zhao Y, Yu S, Zhao H, Li L, Li Y, Liu M, Jiang L 2. 2023. Integrated multi-omics analysis reveals the positive leverage of citrus flavonoids on hindgut microbiota and host homeostasis by modulating sphingolipid metabolism in mid-lactation dairy cows consuming a high-starch diet. Microbiome 11:236. doi:10.1186/s40168-023-01661-4 37880759 PMC10598921

[9] Guo J, Mu R, Li S, Zhang N, Fu Y, Hu X. 2021. Characterization of the bacterial community of rumen in dairy cows with laminitis. Genes (Basel) 12:1996. doi:10.3390/genes12121996 34946945 PMC8700892

[10] Tong J, Zhang H, Zhang Y, Xiong B, Jiang L. 2019. Microbiome and metabolome analyses of milk from dairy cows with subclinical Streptococcus agalactiae mastitis-potential biomarkers. Front Microbiol 10:2547. doi:10.3389/fmicb.2019.02547 31781063 PMC6851174

[11] Jost T, Lacroix C, Braegger CP, Rochat F, Chassard C. 2014. Vertical mother-neonate transfer of maternal gut bacteria via breastfeeding. Environ Microbiol 16:2891–2904. doi:10.1111/1462-2920.12238 24033881

[12] Zhao C, Hu X, Qiu M, Bao L, Wu K, Meng X, Zhao Y, Feng L, Duan S, He Y, Zhang N, Fu Y. 2023. Sialic acid exacerbates gut dysbiosis-associated mastitis through the microbiota-gut-mammary axis by fueling gut microbiota disruption. Microbiome 11:78. doi:10.1186/s40168-023-01528-8 37069691 PMC10107595

[13] Ma C, Sun Z, Zeng B, Huang S, Zhao J, Zhang Y, Su X, Xu J, Wei H, Zhang H. 2018. Cow-to-mouse fecal transplantations suggest intestinal microbiome as one cause of mastitis. Microbiome 6:200. doi:10.1186/s40168-018-0578-1 30409169 PMC6225715

[14] Chuang S, Li K, Tu P, Ho S, Hsu C, Hsieh J, Chen M. 2021. Investigating the reciprocal interrelationships among the ruminal microbiota, metabolome, and mastitis in early lactating holstein dairy cows. Animals (Basel) 11:3108. doi:10.3390/ani11113108 34827839 PMC8614428

[15] Shi W, Moon CD, Leahy SC, Kang D, Froula J, Kittelmann S, Fan C, Deutsch S, Gagic D, Seedorf H, Kelly WJ, Atua R, Sang C, Soni P, Li D, Pinares-Patiño CS, McEwan JC, Janssen PH, Chen F, Visel A, Wang Z, Attwood GT, Rubin EM. 2014. Methane yield phenotypes linked to differential gene expression in the sheep rumen microbiome. Genome Res 24:1517–1525. doi:10.1101/gr.168245.113 24907284 PMC4158751

[16] Roehe R, Dewhurst RJ, Duthie CA, Rooke JA, McKain N, Ross DW, Hyslop JJ, Waterhouse A, Freeman TC, Watson M, Wallace RJ. 2016. Bovine host genetic variation influences rumen microbial methane production with best selection criterion for low methane emitting and efficiently feed converting hosts based on metagenomic gene abundance. PLoS Genet 12:e1005846. doi:10.1371/journal.pgen.1005846 26891056 PMC4758630

[17] Stewart RD, Auffret MD, Warr A, Walker AW, Roehe R, Watson M. 2019. Compendium of 4,941 rumen metagenome-assembled genomes for rumen microbiome biology and enzyme discovery. Nat Biotechnol 37:953–961. doi:10.1038/s41587-019-0202-3 31375809 PMC6785717

[18] Seshadri R, Leahy SC, Attwood GT, Teh KH, Lambie SC, Cookson AL, Eloe-Fadrosh EA, Pavlopoulos GA, Hadjithomas M, Varghese NJ, Paez-Espino D, Perry R, Henderson G, Creevey CJ, Terrapon N, Lapebie P, Drula E, Lombard V, Rubin E, Kyrpides NC, Henrissat B, Woyke T, Ivanova NN, Kelly WJ, Hungate1000 project collaborators. 2018. Cultivation and sequencing of rumen microbiome members from the Hungate1000 collection. Nat Biotechnol 36:359–367. doi:10.1038/nbt.4110 29553575 PMC6118326

[19] Xue MY, Wu JJ, Xie YY, Zhu SL, Zhong YF, Liu JX, Sun HZ. 2022. Investigation of fiber utilization in the rumen of dairy cows based on metagenome-assembled genomes and single-cell RNA sequencing. Microbiome 10:11. doi:10.1186/s40168-021-01211-w 35057854 PMC8772221

[20] Xue MY, Xie YY, Zhong Y, Ma XJ, Sun HZ, Liu JX. 2022. Integrated meta-omics reveals new ruminal microbial features associated with feed efficiency in dairy cattle. Microbiome 10:32. doi:10.1186/s40168-022-01228-9 35172905 PMC8849036

[21] Hu X, Li S, Mu R, Guo J, Zhao C, Cao Y, Zhang N, Fu Y. 2022. The rumen microbiota contributes to the development of mastitis in dairy cows. Microbiol Spectr 10:e02512-21. doi:10.1128/spectrum.02512-21 35196821 PMC8865570

[22] Rodríguez JM, Fernández L, Verhasselt V. 2021. The gut‒breast axis: programming health for life. Nutrients 13:606. doi:10.3390/nu13020606 33673254 PMC7917897

[23] Wang J, Zhang X, He X, Yang B, Wang H, Shan X, Li C, Sun D, Wu R. 2018. LPS-induced reduction of triglyceride synthesis and secretion in dairy cow mammary epithelial cells via decreased SREBP1 expression and activity. J Dairy Res 85:439–444. doi:10.1017/S0022029918000547 30088470

[24] Franzoi M, Manuelian CL, Penasa M, De Marchi M. 2020. Effects of somatic cell score on milk yield and mid-infrared predicted composition and technological traits of Brown Swiss, Holstein Friesian, and Simmental cattle breeds. J Dairy Sci 103:791–804. doi:10.3168/jds.2019-16916 31733847

[25] Bogado Pascottini O, Bruinjé TC, Couto Serrenho R, Mion B, LeBlanc SJ. 2021. Association of metabolic markers with neutrophil function in healthy postpartum dairy cows. Vet Immunol Immunopathol 232:110182. doi:10.1016/j.vetimm.2020.110182 33401107

[26] Cônsolo NRB, Munro JC, Bourgon SL, Karrow NA, Fredeen AH, Martell JE, Montanholi YR. 2018. Associations of blood analysis with feed efficiency and developmental stage in grass-fed beef heifers. Animals (Basel) 8:133. doi:10.3390/ani8080133 30072590 PMC6116025

[27] Li Y, Jiang X, Xu H, Lv J, Zhang G, Dou X, Zhang Y, Li X. 2020. Acremonium terricola culture plays anti-inflammatory and antioxidant roles by modulating MAPK signaling pathways in rats with lipopolysaccharide-induced mastitis. Food Nutr Res 13:64. doi:10.29219/fnr.v64.3649 PMC7681784 33281536

[28] Zhao X, Lacasse P. 2008. Mammary tissue damage during bovine mastitis: causes and control. J Anim Sci 86:57–65. doi:10.2527/jas.2007-0302 17785603

[29] Dai D, Kong F, Han H, Shi W, Song H, Yoon I, Wang S, Liu X, Lu N, Wang W, Li S. 2024. Effects of postbiotic products from Saccharomyces cerevisiae fermentation on lactation performance, antioxidant capacity, and blood immunity in transition dairy cows. J Dairy Sci 107:10584–10598. doi:10.3168/jds.2023-24435 39004128

[30] Guo W, Liu J, Li W, Ma H, Gong Q, Kan X, Cao Y, Wang J, Fu S 2. 2020. Niacin alleviates dairy cow mastitis by regulating the GPR109A/AMPK/NRF2 signaling pathway. Int J Mol Sci 21:3321. doi:10.3390/ijms21093321 32397071 PMC7246865

[31] Tong J, Zhang H, Yang D, Zhang Y, Xiong B, Jiang L. 2018. Illumina sequencing analysis of the ruminal microbiota in high-yield and low-yield lactating dairy cows. Plos one 13:e0198225. doi:10.1371/journal.pone.0198225 30423588 PMC6234037

[32] Wang Y, Nan X, Zhao Y, Jiang L, Wang M, Wang H, Zhang F, Xue F, Hua D, Liu J, Yao J, Xiong B. 2021. Rumen microbiome structure and metabolites activity in dairy cows with clinical and subclinical mastitis. J Anim Sci Biotechnol 12:36. doi:10.1186/s40104-020-00543-1 33557959 PMC7869221

[33] Zhong Y, Xue M, Liu J. 2018. Composition of rumen bacterial community in dairy cows with different levels of somatic cell counts. Front Microbiol 9:3217. doi:10.3389/fmicb.2018.03217 30619238 PMC6312127

[34] Wang Y, Zhao Y, Tang X, Nan X, Jiang L, Wang H, Liu J, Yang L, Yao J, Xiong B. 2024. Nutrition, gastrointestinal microorganisms and metabolites in mastitis occurrence and control. Anim Nutr 17:220–231. doi:10.1016/j.aninu.2024.01.010 38800734 PMC11126769

[35] Lam S, Munro JC, Zhou M, Guan LL, Schenkel FS, Steele MA, Miller SP, Montanholi YR. 2018. Associations of rumen parameters with feed efficiency and sampling routine in beef cattle. Animal 12:1442–1450. doi:10.1017/S1751731117002750 29122053

[36] Metzler-Zebeli BU, Haselmann A, Klevenhusen F, Knaus W, Zebeli Q. 2018. Lactic acid treatment of by-products and phosphorus level in the diet modulate bacterial microbiome and the predicted metagenome functions using the rumen simulation technique. J Dairy Sci 101:9800–9814. doi:10.3168/jds.2018-14821 30146296

[37] Memon MA, Wang Y, Xu T, Ma N, Zhang H, Roy AC, Aabdin ZU, Shen X. 2019. Lipopolysaccharide induces oxidative stress by triggering MAPK and Nrf2 signalling pathways in mammary glands of dairy cows fed a high-concentrate diet. Microb Pathog 128:268–275. doi:10.1016/j.micpath.2019.01.005 30630066

[38] Lück R, Deppenmeier U. 2022. Genetic tools for the redirection of the central carbon flow towards the production of lactate in the human gut bacterium Phocaeicola (Bacteroides) vulgatus. Appl Microbiol Biotechnol 106:1211–1225. doi:10.1007/s00253-022-11777-6 35080666 PMC8816746

[39] Gharechahi J, Vahidi MF, Bahram M, Han JL, Ding XZ, Salekdeh GH. 2021. Metagenomic analysis reveals a dynamic microbiome with diversified adaptive functions to utilize high lignocellulosic forages in the cattle rumen. ISME J 15:1108–1120. doi:10.1038/s41396-020-00837-2 33262428 PMC8114923

[40] Xue MY, Sun HZ, Wu XH, Liu JX, Guan LL. 2020. Multi-omics reveals that the rumen microbiome and its metabolome together with the host metabolome contribute to individualized dairy cow performance. Microbiome 8:64. doi:10.1186/s40168-020-00819-8 32398126 PMC7218573

[41] Sun HZ, Zhou M, Wang O, Chen Y, Liu JX, Guan LL. 2020. Multi-omics reveals functional genomic and metabolic mechanisms of milk production and quality in dairy cows. Bioinformatics 36:2530–2537. doi:10.1093/bioinformatics/btz951 31873721

[42] Liu H, Hu L, Han X, Zhao N, Xu T, Ma L, Wang X, Zhang X, Kang S, Zhao X, Xu S. 2020. Tibetan sheep adapt to plant phenology in alpine meadows by changing rumen microbial community structure and function. Front Microbiol 11:587558. doi:10.3389/fmicb.2020.587558 33193243 PMC7649133

[43] Sibai M, Altuntaş E, Yıldırım B, Öztürk G, Yıldırım S, Demircan T. 2020. Microbiome and longevity: high abundance of longevity-linked muribaculaceae in the gut of the long-living rodent. OMICS 24:592–601. doi:10.1089/omi.2020.0116 32907488

[44] Chen H, Liu Y, Huang K, Yang B, Zhang Y, Yu Z, Wang J. 2022. Fecal microbiota dynamics and its relationship to diarrhea and health in dairy calves. J Anim Sci Biotechnol 13:132. doi:10.1186/s40104-022-00758-4 36307885 PMC9616619

[45] Shen J, Zheng L, Chen X, Han X, Cao Y, Yao J. 2020. Metagenomic analyses of microbial and carbohydrate-active enzymes in the rumen of dairy goats fed different rumen degradable starch. Front Microbiol 11:1003. doi:10.3389/fmicb.2020.01003 32508797 PMC7251062

[46] Wang Y, Nan X, Zhao Y, Jiang L, Wang H, Zhang F, Hua D, Liu J, Yao J, Yang L, Luo Q, Xiong B. 2021. Dietary supplementation of inulin ameliorates subclinical mastitis via regulation of rumen microbial community and metabolites in dairy cows. Microbiol Spectr 9:e00105-21. doi:10.1128/Spectrum.00105-21 34494854 PMC8557905

[47] Hoque MN, Istiaq A, Rahman MS, Islam MR, Anwar A, Siddiki A, Sultana M, Crandall KA, Hossain MA. 2020. Microbiome dynamics and genomic determinants of bovine mastitis. Genomics 112:5188–5203. doi:10.1016/j.ygeno.2020.09.039 32966856

[48] Giustarini D, Colombo G, Garavaglia ML, Astori E, Portinaro NM, Reggiani F, Badalamenti S, Aloisi AM, Santucci A, Rossi R, Milzani A, Dalle-Donne I. 2017. Assessment of glutathione/glutathione disulphide ratio and S-glutathionylated proteins in human blood, solid tissues, and cultured cells. Free Radic Biol Med 112:360–375. doi:10.1016/j.freeradbiomed.2017.08.008 28807817

[49] Zhang H, Li H, Pan B, Zhang S, Su X, Sun W, Zhang T, Zhang Z, Lv S, Cui H. 2023. Polygonatum sibiricum integrated 16S rRNA sequencing and untargeted metabolomics analysis to reveal the protective mechanisms of Polysaccharide on Type 2 diabetes mellitus model rats. Curr Drug Metab 24:270–282. doi:10.2174/1389200224666230406114012 37038712

[50] Lu Z, He X, Ma B, Zhang L, Li J, Jiang Y, Zhou G, Gao F. 2019. Dietary taurine supplementation improves breast meat quality in chronic heat-stressed broilers via activating the Nrf2 pathway and protecting mitochondria from oxidative attack. J Sci Food Agric 99:1066–1072. doi:10.1002/jsfa.9273 30014460

[51] Thomas FC, Mudaliar M, Tassi R, McNeilly TN, Burchmore R, Burgess K, Herzyk P, Zadoks RN, Eckersall PD. 2016. Mastitomics, the integrated omics of bovine milk in an experimental model of Streptococcus uberis mastitis: 3. untargeted metabolomics. Mol Biosyst 12:2762–2769. doi:10.1039/c6mb00289g 27412568

[52] Tavella T, Rampelli S, Guidarelli G, Bazzocchi A, Gasperini C, Pujos-Guillot E, Comte B, Barone M, Biagi E, Candela M, Nicoletti C, Kadi F, Battista G, Salvioli S, O’Toole PW, Franceschi C, Brigidi P, Turroni S, Santoro A. 2021. Elevated gut microbiome abundance of Christensenellaceae, Porphyromonadaceae and Rikenellaceae is associated with reduced visceral adipose tissue and healthier metabolic profile in Italian elderly. Gut Microbes 13:1–19. doi:10.1080/19490976.2021.1880221 PMC7889099 33557667

[53] Zhao Y, Zhao H, Li L, Tan J, Wang Y, Liu M, Jiang L. 2023. Multi-omics analysis reveals that the metabolite profile of raw milk is associated with dairy cows’ health status. Food Chem 428:1–12. doi:10.1016/j.foodchem.2023.136813 37421666

[54] Cao Y, Wang D, Wang L, Wei X, Li X, Cai C, Lei X, Yao J. 2021. Physically effective neutral detergent fiber improves chewing activity, rumen fermentation, plasma metabolites, and milk production in lactating dairy cows fed a high-concentrate diet. J Dairy Sci 104:5631–5642. doi:10.3168/jds.2020-19012 33663818

[55] Xie F, Jin W, Si H, Yuan Y, Tao Y, Liu J, Wang X, Yang C, Li Q, Yan X, Lin L, Jiang Q, Zhang L, Guo C, Greening C, Heller R, Guan LL, Pope PB, Tan Z, Zhu W, Wang M, Qiu Q, Li Z, Mao S. 2021. An integrated gene catalog and over 10,000 metagenome-assembled genomes from the gastrointestinal microbiome of ruminants. Microbiome 9:137. doi:10.1186/s40168-021-01078-x 34118976 PMC8199421

