# Supplementary material for: Rumen microbes affect the somatic cell counts of dairy cows by modulating glutathione metabolism
Source: mSystems. 2025 Mar 19;10(4):e01093-24. doi: 10.1128/msystems.01093-24 (PMC12013278; doi:10.1128/msystems.01093-24)
Supplement: Supplemental figures — Figures S1 to S3. [file msystems.01093-24-s0001.docx]

Supplemental Legends


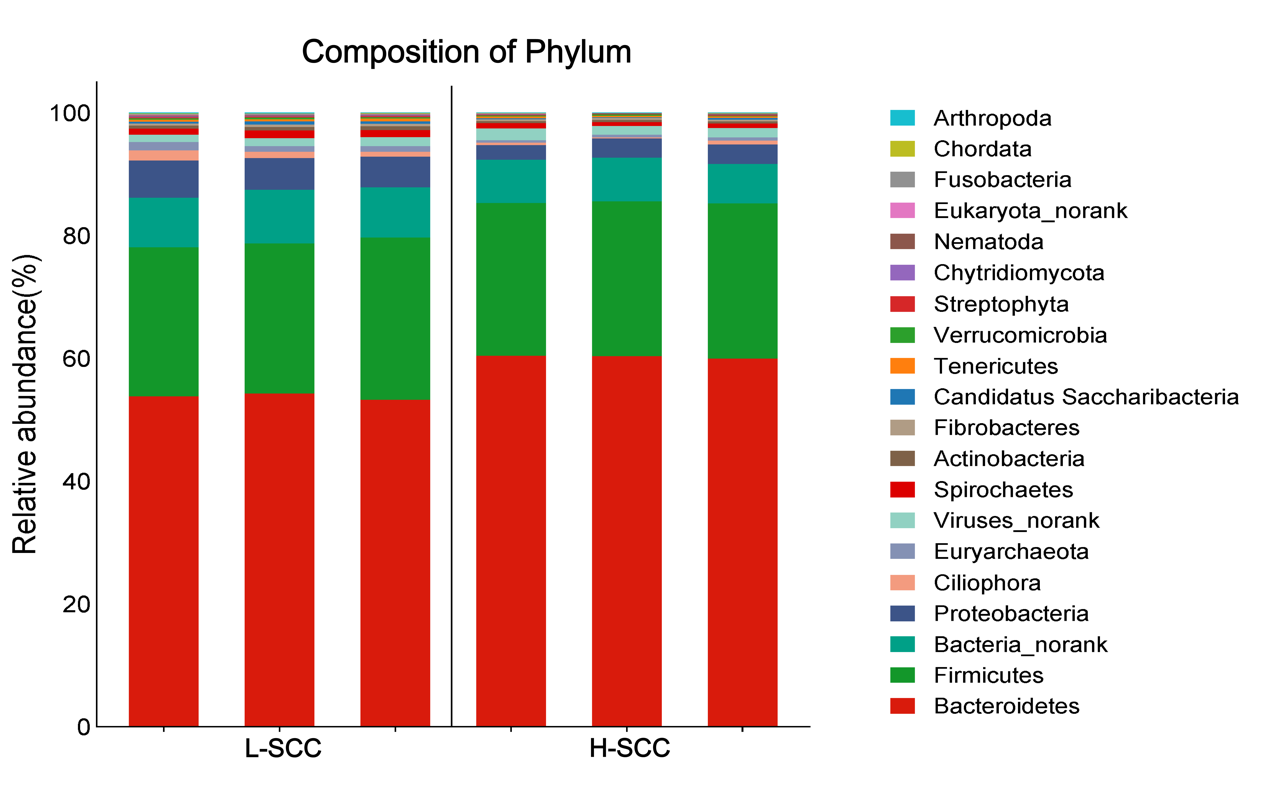


Supplemental Figure1. Bacterial Community Composition at the Phylum Level Analyzed by Metagenomics.


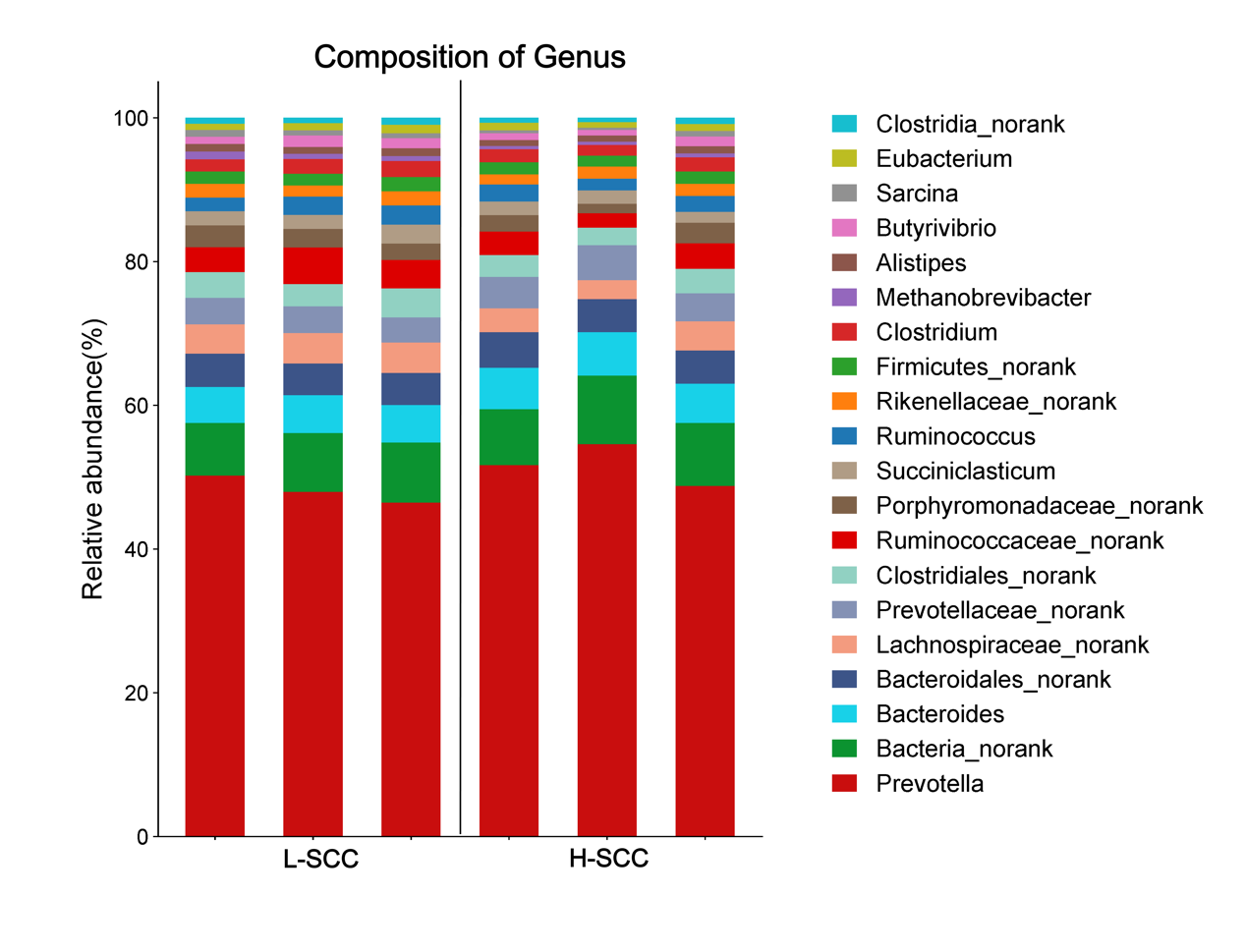


Supplemental Figure2. Bacterial Community Composition at the Genus Level Analyzed by Metagenomics.


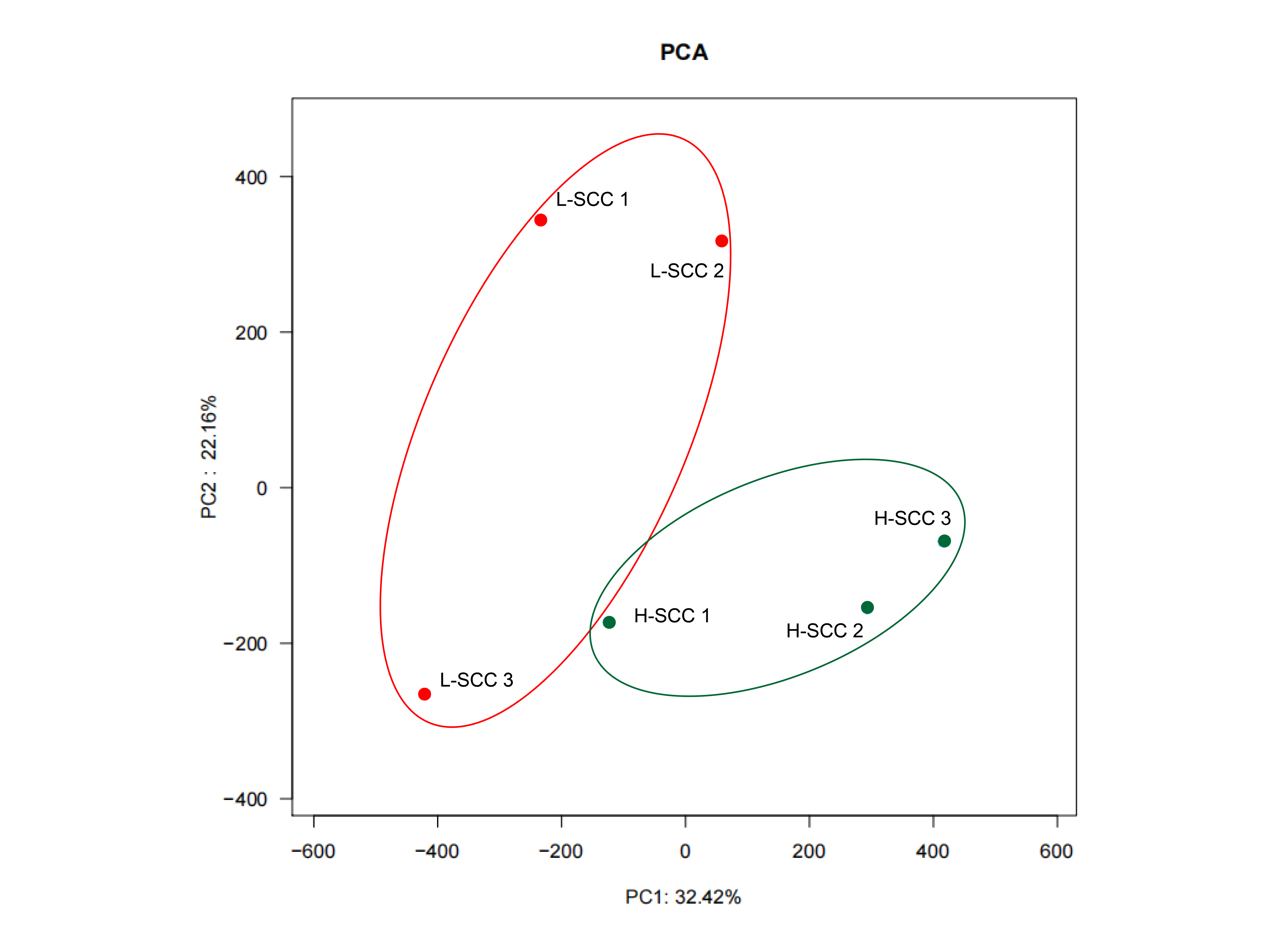


Supplemental Figure3. Multi-Sample PCA Analysis of Differences in Rumen Microbial Composition
